# The Principals Underlying the Clamp and Operation for Hemorrhoids

**Published:** 1917-12

**Authors:** W. Oakley Hermance

**Affiliations:** Philadelphia, Pa.


### THE PRINCIPLES UNDERLYING THE CLAMP AND OPERATION FOR HEMORRHOIDS.

**By W. Oakley Hermance, M.D., Philadelphia, Pa.**

The author calls attention to the purpose of this form of operation.

First: To remove actual piles or pathology.

Second: To support relaxed bearing tissue and mucous membrane. After giving minute details as to his technic he insists that only just ENOUGH tissue be removed to care for the pathology, being sure that columns of mucosa and skin be left between the eschare, thus preventing any undue contraction of the rectal outlet. Unless the tissue included in the clamp is excessive it is not cut off but is destroyed by the careful application of the cautery.

Care is taken that multiple strips of wet gauze are placed under the clamp to prevent undue radication of heat to the surrounding tissues. Careful placing of the clamp, thorough cooking of the inclined tissues combined with the onrushing produced by the clamp is depended upon to prevent such a complication as homorrhage. After all danger of secondary hemorrhage has passed the insertion of a gloved finger will overcome the tendency to contraction.

